# Asymmetric Transfer Hydrogenation of Arylketones Catalyzed by Enantiopure Ruthenium(II)/Pybox Complexes Containing Achiral Phosphonite and Phosphinite Ligands

**DOI:** 10.3390/molecules25040990

**Published:** 2020-02-23

**Authors:** Miguel Claros, Eire de Julián, Josefina Díez, Elena Lastra, M. Pilar Gamasa

**Affiliations:** Departamento de Química Orgánica e Inorgánica-IUQOEM (Unidad Asociada al CSIC), Centro de Innovación en Química Avanzada (ORFEO-CINQA), Universidad de Oviedo, E-33006 Oviedo, Principado de Asturias, Spain; clarosca.mi@gmail.com (M.C.); eiredejulian@gmail.com (E.d.J.); jdv@uniovi.es (J.D.); elb@uniovi.es (E.L.)

**Keywords:** asymmetric catalysis, transfer hydrogenation, alcohols, ruthenium, pybox, phosphonite ligands, phosphinite ligands

## Abstract

A family of complexes of the formula *trans*-[RuCl_2_(L)(R-pybox)] (R-pybox = (*S*,*S*)-*^i^*Pr-pybox, (*R*,*R*)-Ph-pybox, L = monodentate phosphonite, PPh(OR)_2_, and phosphinite, L = PPh_2_(OR), ligands) were screened in the catalytic asymmetric transfer hydrogenation of acetophenone, observing a strong influence of the nature of both the R-pybox substituents and the L ligand in the process. The best results were obtained with complex *trans*-[RuCl_2_{PPh_2_(OEt)}{(*R*,*R*)-Ph-pybox}] (**2c**), which provided high conversion and enantioselectivity (up to 96% enantiomeric excess, *e.e.*) for the reduction of a variety of aromatic ketones, affording the (*S*)-benzylalcohols.

## 1. Introduction

**Figure 1:**
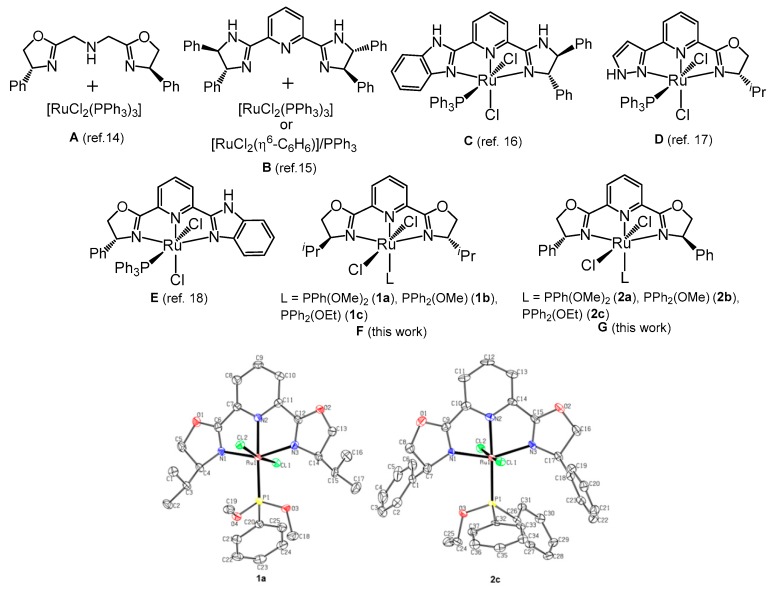
Selected ruthenium(II) complexes, bearing enantiopure *C*_2_ or *C*_1_ symmetry *N*,*N*,*N*-donor ligands, used in asymmetric transfer hydrogenation (ATH) of ketones (Examples corresponding to complexes **A**–**E** feature the N-H functionality; structures **F**–**G** lack the N-H functionality).

Asymmetric transfer hydrogenation (ATH) of prochiral ketones catalyzed by transition metal complexes, displaying well designed chiral ligands, is currently recognized as a powerful and versatile tool to access enantiopure alcohols [1,2,3,4,5,6,7,8,9,10,11,12,13]. In this area, it must be mentioned that the studies focused on the asymmetric transfer hydrogenation (ATH) of ketones using ruthenium(II) complexes bearing PPh_3_ and enantiopure *C*_2_ or *C*_1_ symmetry *N*,*N*,*N*-donor ligands with NH functionality (see Figure 1A–E). Thus, the groups of Zhang [14] and Beller [15] described the use of in situ-generated ruthenium complexes, containing bis(oxazolinylmethyl)amine (ambox ligand) (Figure 1A) [14] and pyridine(bis)imidazolines (pybim ligand) (Figure 1B) [15], respectively. On the other hand, it has also been reported that isolated ruthenium complexes with *C*_1_-symmetry *N*,*N*,*N* donor ligands (Figure 1C–E) [16,17,18] efficiently catalyze the asymmetric transfer hydrogenation of aryl ketones. In all of these examples, the presence of the NH functionality in the enantiopure ligand is a requisite for achieving high reactivity and enantioselectivity.

We have also made some contributions in this field, particularly on the synthesis and catalytic activity of group 8 metal complexes based on enantiopure *N,N,N*-ligands, lacking the N-H functionality. In this context, we reported the complexes *cis*-[MCl_2_(L)(R-pybox)] (M = Ru, Os, L = phosphane) and *trans*-[MCl_2_(L)(R-pybox)] (M = Ru, L = phosphane, phosphite; M = Os, L = phosphite) and studied their catalytic activity toward the asymmetric transfer hydrogenation of aryl ketones. Interestingly, the efficiency of these catalysts depends not only on the metal and the chiral ligand but also on the nature of the auxiliary P-donor ligand [19,20]. Thus, we found that the reduction of different aryl ketones was best achieved using the combination of Ru(II)/(*R,R*)-Ph-pybox/phosphane (95%–99% conversion; up to 95% *e.e.*) and Os(II)/(*S,S*)-*^i^*Pr-pybox)/phosphite (96%–99% conversion; up to 94% *e.e.*). However, the corresponding phosphite ruthenium and phosphane osmium complexes led to lower enantiomeric excess. The influence of the P-donor ligand was also analyzed by Beller and col. using mixtures of [RuCl_2_(*η*^6^-C_6_H_6_)]/pybim and monodentate or bidentate phosphanes as the catalyst [15]. In the light of these findings, we found of interest to move to explore further combinations of ruthenium/osmium complexes with other phosphorous ligands with different electronic demand and steric requirement.

Continuing our studies on the ATH of ketones using ruthenium(II) complexes, containing pybox derivatives as the chiral auxiliary and phosphane/phosphite as the achiral phosphorous ligand, we reported the ATH reaction of aryl ketones catalyzed by ruthenium complexes *trans*-[RuCl_2_(L){(*S,S*)-*^i^*Pr-pybox}] and *trans*-[RuCl_2_(L){(*R*,*R*)-Ph-pybox}], containing monodentate phosphonite (L = PPh(OR)_2_) and phosphinite (L = PPh_2_(OR)) ligands (Figure 1F,G) [21]. Such ligands can be placed between phosphanes and phosphites in a donor-acceptor ligand scale.

## 2. Results and Discussion

### 2.1. Ruthenium(II)/R-pybox Catalysts **1a**–**1c** and **2a**–**2c**

We explored the effectiveness of ruthenium(II) complexes **1**–**2** (Figure 1F,G), containing achiral phosphonite (**1a, 2a**) and phosphinite (**1b,c**, **2b,c**) ligands [21], in the ATH reaction of aryl ketones. In accordance with their *C*_2_ symmetric structure, complexes **1**–**2** showed a single set of signals for the two oxazoline fragments in the ^1^H and ^13^C{^1^H} NMR spectra. Therefore, the chloro ligands and the L/pyridine ligands adopted a trans-arrangement.

Moreover, this stereochemistry had been confirmed by single-crystal X-ray analysis for complexes **1a** and **2c**. An ORTEP-type view of these complexes is shown in Figure 1 (thermal ellipsoids were shown at the 30% probability level, and hydrogen atoms were omitted for clarity), and selected bonding data are collected in the Table S1 (Supporting Information).

Both structures exhibited a distorted octahedral geometry around the ruthenium atom, which was bonded to the three nitrogen atoms (R-pybox; R = *^i^*Pr (**1a**), Ph (**2c**)), to the phosphorous atom (PPh(OMe)_2_ (**1a**), PPh_2_(OEt) (**2c**)), and to two chlorine atoms. The chlorine atoms were located in a *trans* disposition with Cl(1)-Ru(1)-Cl(2) angles of 171.60(6) (**1a**) and 173.25(5)° (**2c**). The N(2)–Ru(1)–P(1) angle was close to the linearity (178.3(2) (**1a**) and 177.76(15)° (**2c**)), and the Ru(1)–P(1) distances were 2.2625(18) (**1a**) and 2.2829(13) (**2c**) Å. The Ru(1)–N(1), Ru(1)–N(2), and Ru(1)–N(3) distances, as well as the N–Ru(1)–N bond angles, fell in the range observed for other related ruthenium(II) pybox complexes [22,23] (see Table S1, Supporting Information).

### 2.2. Catalytic Asymmetric Transfer Hydrogenation of Ketones

Then, the above-reported complexes were tested towards the ATH of acetophenone. Thus, Table 1 summarizes the conversion of acetophenone into enriched 1-phenylethanol using the ruthenium(II) (*S,S*)-*^i^*Pr-pybox) (**1**) and (*R,R*)-Ph-pybox (**2**) catalysts. The electronic nature and steric demand [24,25] (Tolman electronic parameter (TEP) and cone angles) of the achiral auxiliary phosphonite and phosphinite ligands used in this report were between those of the phosphane and phosphite ligands, already studied by us in the ATH of ketones [19].

In a typical experiment, the ruthenium catalyst precursor **1**,**2** (0.01 mmol, 0.2 mol%) was added to a solution containing 5 mmol of acetophenone in 45 mL of 2-propanol under an argon atmosphere, and the solution was stirred initially for 15 min at 82 °C. After this time, a solution of 0.24 mmol of KO*^t^*Bu in 5 mL of 2-propanol (ketone/catalyst/KO*^t^*Bu ratio = 500:1:24) was added, and the resulting mixture was heated for 15 min and then monitored by gas chromatography using a chromatograph with a Supelco *β*-DEX 120 chiral capillary column (90 min of stirring time was used in some instances; see Table 1, entries 2,6,8).

From the results displayed in Table 1, several features worth attention: i) All of the complexes were active towards the asymmetric reduction of acetophenone, the conversion varying from moderate (48%–67%, entries 1,5,7) to high (95%–96%, entries 3,4,9) after 15 min of reaction. ii) The efficiency of the catalyst depended greatly on the auxiliary achiral P-ligand (phosphonite vs. phosphinite). Thus, the phosphinite complexes **1b**–**c**/**2b**–**c** were notably superior to the phosphonite complexes **1a**/**2a** (entries 3,4,7,8,9 vs. 1,2,5,6). The values of asymmetric induction obtained were in accordance with the Tolman electronic parameter (cm^−1^) and the cone angles (deg) of these achiral ligands [24,25]. iii) Complexes **1** and **2** were able to selectively convert acetophenone into (*R*)- and (*S*)-1-phenylethanol, respectively. Under these conditions tested, the chiral ligand did not significantly affect the efficiency of the reaction (*^i^*Pr-pybox, entries 1–4; Ph-pybox, entries 5–9).

Therefore, once the basic reaction conditions were defined, we carried out the optimization of the reduction of acetophenone using the complex [RuCl_2_{PPh_2_(OEt)}(Ph-pybox)] (**2c**) as catalyst (Table 2). The following parameters were explored: i) Catalyst loading (entries 1–4): First of all, the amount of catalyst was varied in the range of 0.1–0.4 mol% (entries 1–4), starting from the basic reaction conditions (entry 1; ketone/KO*^t^*Bu ratio of 500:24; 50 mL of 2-propanol). The highest e.e. value was obtained when 0.3 mol% of catalyst was employed (entry 3). Lower ratio of catalyst (0.2 and 0.1 mol%; entries 1–2) required longer reaction times and/or led to poorer enantiomeric excess, while a little decrease of the *e.e.* was observed with 0.4 mol% of catalyst (entry 4). ii) Concentration (entries 5, 6): Then, we studied the effect of the amount of 2-propanol and found a notable improvement of both conversion and enantioselectivity as the total amount of 2-propanol increased from 50 mL (entry 3) to 75 mL (entries 5, 6). iii) Base (entries 5–11): Taking the entries 5, 6 as the reference, some other bases were explored (entries 7–11). We found that KO*^t^*Bu, NaOH, and NaO*^t^*Bu behaved more efficiently than KOH and Cs_2_CO_3_ (entries 5–9 vs. 10, 11). Among the former, the base NaO*^t^*Bu seemed to be slightly superior (entries: 8, 9 vs. 5, 6). iv) Ketone/catalyst/NaO^t^Bu ratio (entries 8, 12, 13): Decreasing the ratio of base from 500:1.5:24 (entry 8) to either 500:1.5:12 or 500:1.5:6 (entries 12, 13) resulted in a slightly higher *e.e.*

Once the reduction of acetophenone was optimized using **2c** as a catalyst [26], the ATH of a number of aryl ketones was studied, and the results are gathered in Table 3. In most cases, the reaction required short reaction time (5–10 min) and took place with high conversion (around 90%) and excellent enantioselectivity (87%–96% *e.e.*). Thus, this pattern was observed for acetophenone, propiophenone, and 2-acetonaphthone (entries 1, 2 and 3), as well as for electron-rich 3- and 4-methoxyacetophenone (entries 4 and 5) (3000–4000 TOF) [27]. On the other hand, increasing the steric demand of the substrate, particularly that of the aryl group, had a great impact on the process in terms of both conversion and enantiomeric excess. Thus, although the reduction of 2-methoxyacetophenone (entries 6 and 7) was rather inefficient, isobutyrophenone could be transformed into the alcohol with high enantiomeric excess and moderate (entry 8) or good conversion (entry 9).

**Figure 2:**
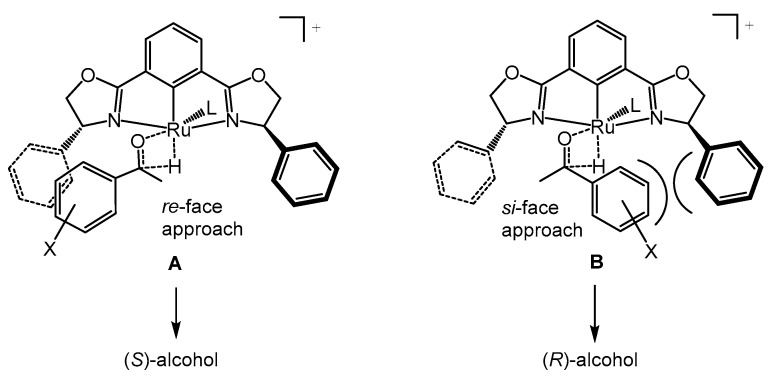
Proposed transition states for the asymmetric transfer hydrogenation of aryl ketones.

Complexes **1** and **2** fulfilled the requirement to be precursors of active species in the catalytic asymmetric transfer hydrogenation of ketones by hydrogen transfer from *^i^*PrOH/base. Regarding the stereochemical outcome, the observed results were in accordance with the established reaction pathway [28,29,30,31]. Thus, the hydrogen transfer step had been proposed to occur through a four-membered cyclic transition state, involving the metal-hydride and the ketone. Such a transition state would result from i) chloride extraction and metal isopropoxide formation, ii) generation of metal hydride by β-elimination of propanone (an inner-sphere mechanism), iii) loss of the second chloride to generate a 16-electron metal hydride, iv) coordination of metal hydride and ketone. Then, the approach of the ketone to the metal-hydride complex could occur in two ways, involving the *re*-face or *si*-face of the ketone. Figure 2 illustrates the transition states in the case of (*R*,*R*)-pybox complexes, showing that the steric repulsions of the oxazoline- and ketone-phenyl groups make transition state **A** lower in energy than transition state **B,** providing the (*S)*-alcohols preferentially [19,32]. Conversely, the use of (*S*,*S*)-pybox complexes favored the formation of the corresponding (*R*)-alcohols.

## 3. Materials and Methods

### 3.1. General Comments

The reactions were performed under an atmosphere of dry argon using vacuum-line and standard Schlenk techniques. The reagents were obtained from commercial suppliers and used without further purification. Solvents were dried by standard methods and distilled under argon before use [19,21]. The precursor complexes *trans*-[RuCl_2_(*η*^2^-C_2_H_4_){(*S*,*S*)-*^i^*Pr-pybox}] and *trans*-[RuCl_2_(*η*^2^-C_2_H_4_){(*R*,*R*)-Ph-pybox}] [33,34] and the complexes **1**-**2 [21]** were prepared by reported methods. The ^1^H, ^13^C{^1^H}, and ^31^P{^1^H} NMR spectra of complexes **1a–c** and **2a–c** were reported in the Supplementary Materials [21].

### 3.2. X-Ray Crystal Structure Determination of Complexes 1a·2CHCl_3_ and 2c·2.5CH_2_Cl_2_

Suitable crystals for X-ray diffraction analysis were obtained by slow diffusion of *n*-hexane into a solution of the complexes in chloroform (**1a**) or dichloromethane (**2c**). The most relevant crystal and refinement data are collected in Table S2 in the Supporting Information.

The diffraction data of both complexes were recorded on an Oxford Diffraction Xcalibur Nova (Agilent, Santa Clara, CA, USA) single-crystal diffractometer, using Cu-Kα radiation (λ = 1.5418 Å). Images were collected at a 62 mm fixed crystal-detector distance, using the oscillation method, with 1° oscillation and variable exposure time per image (6.5–35 s (for **1a**) and 30–110 s (for **2c**)). The data collection strategy was calculated with the program CrysAlis Pro CCD [35]. Data reduction and cell refinement were performed with the program CrysAlis Pro RED [35]. Empirical absorption correction was applied using the SCALE3 ABSPACK algorithm, as implemented in the program CrysAlis Pro RED [35].

The software package WINGX [36] was used for space-group determination, structure solution, and refinement. The structures were solved by Patterson interpretation and phase expansion using DIRDIF [37]. In the crystals of **1a** and **2c**, 2CHCl_3_ (for **1a**) or 2.5CH_2_Cl_2_ solvent molecules (for **2c**) per unit formula of the complex were present. For complex **2c** also, other highly disordered solvent molecules were found, which were impossible to refine using conventional discrete-atom models. To resolve these issues, the contribution of solvent electron density was removed by SQUEEZE/PLATON [38]. Isotropic least-squares refinement on *F*^2^ using SHELXL2013 [39] was performed. During the final stages of the refinements, all the positional parameters and the anisotropic temperature factors of all the non-H atoms were refined. The H atoms were geometrically placed, riding on their parent atoms with isotropic displacement parameters set to 1.2 times the Ueq of the atoms to which they were attached (1.5 for methyl groups). For both complexes, the maximum residual electron density was located near heavy atoms.

The function minimized was [*w*(*F*o^2^ − *F*c^2^)/*w*(*F*o^2^)]^1/2^, where w = 1/[*σ*^2^(*F*o^2^) + (*aP*)^2^ + *bP*] (a and b values are collected in the Table S2 in the Supporting Information) with σ(*F*o^2^) from counting statistics and P = (Max (*F*o^2^, 0) + 2*F*c^2^)/3. Atomic scattering factors were taken from the International Tables for X-Ray Crystallography [40]. The crystallographic plots were made with PLATON [38].

CCDC 1,545,753 (**1a**·2CHCl_3_) and CCDC 1,545,754 (**2c**·2.5CH_2_Cl_2_) contain the supplementary crystallographic data for this paper. These data could be obtained free of charge from The Cambridge Crystallographic Data Centre via http://www.ccdc.cam.ac.uk/data_request/cif.

### 3.3. General Procedure for Hydrogen Transfer Reactions

The ruthenium catalyst precursor **1**, **2** (0.01 mmol) was placed in a three-bottomed Schlenk flask containing 5 mmol of ketone in 45 mL of 2-propanol under an argon atmosphere. The solution was heated at 82 °C for 15 min, and then a solution of 0.24 mmol of the base in 5 mL of 2-propanol was added, and the reaction mixture stirred for the time given in Table 1, Table 2 and Table 3. The course of the reaction was monitored by gas chromatography using a chromatography Agilent Model HP-6890 equipped with a Supelco β-DEX 120 chiral capillary column. The enantiomeric ratio and the absolute configuration of the major enantiomer were determined by GC (retention times) and optical rotations, respectively, and compared with literature values. The resulting alcohols and the starting ketones were, in all cases, the only products detected.

## 4. Conclusions

The present study showed that complexes featuring the combination Ru(II)/R-pybox [(*S*,*S*)-*^i^*Pr-pybox, (*R*,*R*)-Ph-pybox]/P-donor ligand (methoxy-phosphonites and methoxy- and ethoxy-phosphinites) were active catalysts toward the ATH reaction of arylketones. The best results were obtained using the complex *trans*-[RuCl_2_{PPh_2_(OEt)}{(*R*,*R*)-Ph-pybox}] (**2c**), providing (S)-benzylalcohols in high yield (87%–98 %) and enantioselectivity (up to 96 % *e.e*.). These results were in the range of those previously described using the complexes *cis*- and *trans*-[RuCl_2_(L) {(*R*,*R*)-Ph-pybox}] (L = PPh_3_, P*^i^*Pr_3_) and were superior to those obtained using the complexes with phosphite ligands *trans*-[RuCl_2_(L){(*R*,*R*)-Ph-pybox}] (L= P(OMe)_3_, P(OPh)_3_)). On the other hand, they confirmed that the nature of the achiral P-donor ligand did influence the efficiency of the catalyst. The values of asymmetric induction obtained were in accordance with the Tolman electronic parameter (cm^−1^) and the cone angles (deg) of these achiral ligands. Moreover, this report illustrated the utility of chiral ligands, lacking the N-H functionality, thus complementing previous works of ATH processes based on chiral N-H ligands.

## Figures and Tables

**Table 1:** Asymmetric Transfer Hydrogenation of Acetophenone Catalyzed by Ruthenium(II) Complexes **1a**–**c** and **2a–c.**

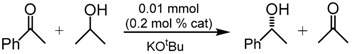				
	Catalyst	Time (min)	Conv. (%) *^a^*	*e.e.* (%) *^a^*
1	[RuCl_2_{PPh(OMe)_2_}(*^i^*Pr-pybox)] (**1a**)	15	63	16 (*R*)
2		90	97	20 (*R*)
3	[RuCl_2_{PPh_2_(OMe)}(*^i^*Pr-pybox)] (**1b**)	15	96	43 (*R*)
4	[RuCl_2_{PPh_2_(OEt)}(*^i^*Pr-pybox)] (**1c**)	15	95	62 (*R*)
5	[RuCl_2_{PPh(OMe)_2_}(Ph-pybox)] (**2a**)	15	67	5 (*S*)
6		90	98	11 (*S*)
7	[RuCl_2_{PPh_2_(OMe)}(Ph-pybox)] (**2b**)	15	48	46 (*S*)
8		90	92	46 (*S*)
9	[RuCl_2_{PPh_2_(OEt)}(Ph-pybox)] (**2c**)	15	95	63 (*S*)

*^a^* Determined by GC with a Supelco *β*-DEX 120 chiral capillary column.

**Table 2:** Optimization of Reaction Conditions for Transfer Hydrogenation of Acetophenone Catalyzed by Ruthenium Complex [RuCl_2_{PPh_2_(OEt)}(Ph-pybox)] (**2c**) *^a^*.

	Catalyst Loading (mol %)	^i^PrOH (mL)	Base	Ketone/BaseRatio	Time (min)	Conversion (%) *^b^*	*e.e.* (%) (*S*) *^b^*
1	0.2	50	KO*^t^*Bu	500/24	15	95	63
2	0.1	50	KO*^t^*Bu	500/24	30	79	67
3	0.3	50	KO*^t^*Bu	500/24	15	91	70
4	0.4	50	KO*^t^*Bu	500/24	15	93	66
5	0.3	75	KO*^t^*Bu	500/24	5	91	87
6	0.3	75	KO*^t^*Bu	500/24	15	97	83
7	0.3	75	NaOH	500/24	10	95	83
8	0.3	75	NaO*^t^*Bu	500/24	5	97	89
9	0.3	75	NaO*^t^*Bu	500/24	10	97	86
10	0.3	75	KOH	500/24	30	95	67
11	0.3	75	Cs_2_CO_3_	500/24	30	95	78
12	0.3	75	NaO*^t^*Bu	500/12	5	96	92
13	0.3	75	NaO*^t^*Bu	500/6	5	97	90

*^a^* Reactions were carried out at 82 °C using 5 mmol of acetophenone. *^b^* Determined by GC with a Supelco *β*-DEX 120 chiral capillary column.

**Table 3:** Transfer Hydrogenation of Ketones Catalyzed by Complex [RuCl_2_{PPh_2_(OEt)}(Ph-pybox)] (**2c**) under Optimized Conditions *^a^*.

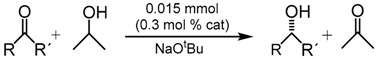					
	Ketone	Time (min)	TOF (h^−1^) *^b^*	Conversion (%) *^c^*	*e.e.* (%) (*S*) *^c^*
1	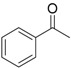	5	3840	96	92
2	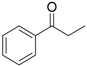	10	3040	97	96
3	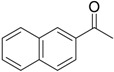	5	3840	96	87
4	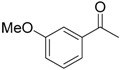	5	3920	98	90
5	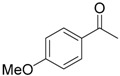	5	3440	86	89
6	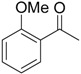	45	500	54	72
7		90	500	75	68
8	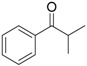	10	720	51	91
9		60	720	90	87

*^a^* Reactions were carried out at 82 °C using 5 mmol of ketone and 2-propanol (75 mL), 0.015 mmol (0.3 mol %) catalyst, and NaO*^t^*Bu (acetophenone/catalyst/NaO*^t^*Bu 500:1.5:12). *^b^* Calculated TOF (turnover frequency) values at t = 5 min. *^c^* Determined by GC with a Supelco *β*-DEX 120 chiral capillary column.

## Supplementary Materials

The following are available online. GC spectra for all the reactions of the Table 1, Table 2 and Table 3. Selected Bond Distances (Å) and Angles (deg) for Complexes **1a**·2CHCl_3_ and **2c**·2.5CH_2_Cl_2_ (Table S1). Crystal Data and Structure Refinement for Complexes **1a**·2CHCl_3_ and **2c**·2.5CH_2_Cl_2_ (Table S2).
