# Supplementary material for: Asymmetric Transfer Hydrogenation of Arylketones Catalyzed by Enantiopure Ruthenium(II)/Pybox Complexes Containing Achiral Phosphonite and Phosphinite Ligands
Source: Molecules. 2020 Feb 23;25(4):990. doi: 10.3390/molecules25040990 (PMC7070392; doi:10.3390/molecules25040990)
Supplement: Supplementary file 1 [file molecules-25-00990-s001.pdf]

Asymmetric Transfer Hydrogenation of Arylketones Catalyzed by Enantiopure Ruhenium(II)/Pybox Complexes Containing Achiral Phosphonite and Phosphinite Ligands

*Miguel Claros, Eire de Julián, Josefina Díez, Elena Lastra, M. Pilar Gamasa**

Departamento de Química Orgánica e Inorgánica-IUQOEM (Unidad Asociada al CSIC), Centro de Innovación en Química Avanzada (ORFEO-CINQA), Universidad de Oviedo, E-33006 Oviedo, Principado de Asturias, Spain.

SUPPORTING INFORMATION

page

1. Enantiomeric excess determined by GC (Table 1)........................S2
2. Enantiomeric excess determined by GC (Table 2)........................S11
3. Enantiomeric excess determined by GC (Table 3)........................S24
4. Table *S*1. Selected Bond Distances (Å) and Angles (deg) for Complexes **1a**·$2CHCl_3$ and **2c**·$2.5CH_2Cl_2$……………………….S33
5. Table *S*2. Crystal Data and Structure Refinement for Complexes **1a**·$2CHCl_3$ and **2c**·$2.5CH_2Cl_2$.................................... S34

Table 1. Asymmetric Transfer Hydrogenation of Acetophenone Catalyzed by Ruthenium(II) Complexes **1a-c** and **2a-c**.

Enantiomeric excess: Determined by GC with a Supelco *β*-DEX 120 chiral capillary column.

Table 1, entry 1

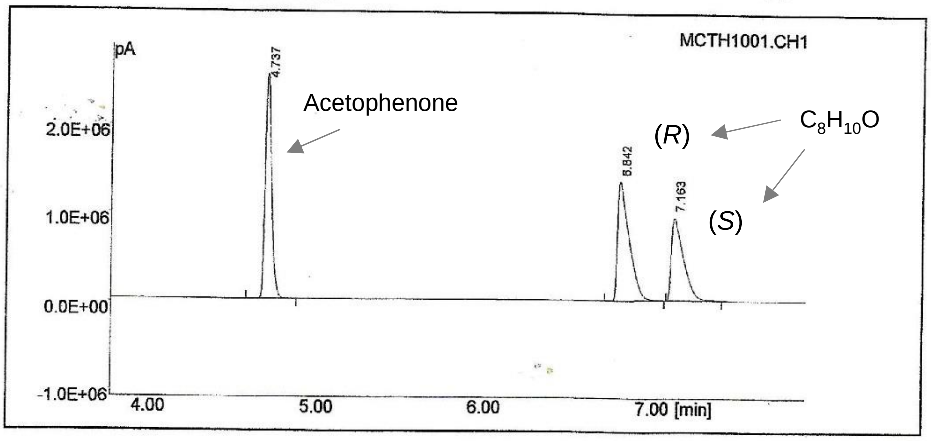

[RuCl$_2${PPh(OMe)$_2$}(^{i}Pr-pybox)] **(1a)**
Acetophenone/**1a**/KO^{t}Bu ratio = 500:1.24, ^{i}PrOH 50 mL
Time of reaction: 15 min

#	Nombre	Tr [min]	Área [pA*s]	%Área
1		4,737	6325275	36,876
2		6,842	6267544	36,540
3		7,163	4559808	26,584

Table 1, entry 2

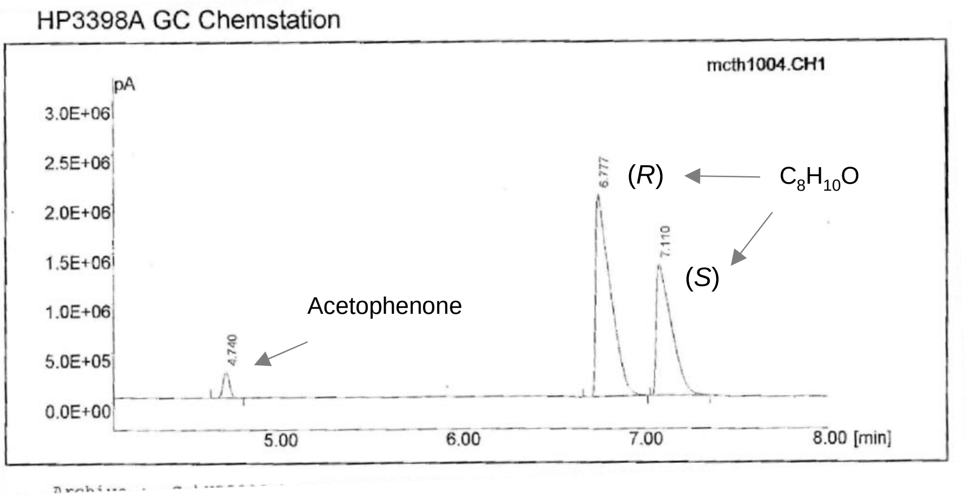

[RuCl$_2${PPh(OMe)$_2$}(^{i}Pr-pybox)] **(1a)**
Acetophenone/**1a**/KO^{t}Bu ratio = 500:1.24, ^{i}PrOH 50 mL
Time of reaction: 90 min

#	Nombre	Tr [min]	Área [pA*s]	%Área
1		4,740	597361	3,169
2		6,777	10946499	58,068
3		7,110	7307277	38,763

Table 1, entry 3

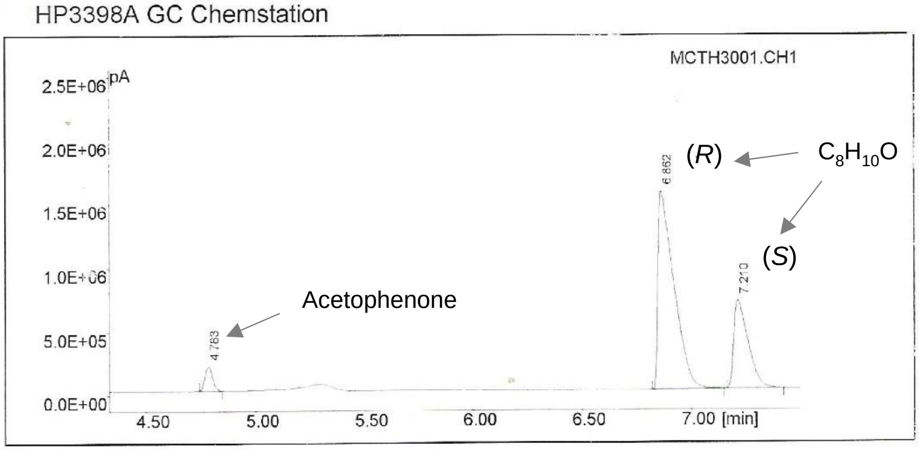

[RuCl$_2${PPh$_2$(OMe)}(^{i}Pr-pybox)] **(1b)**

Acetophenone/**1b**/KO^{t}Bu ratio = 500:1.24, ^{i}PrOH 50 mL

Time of reaction: 15 min

#	Nombre	Tr [min]	Área [pA*s]	%Área
1		4,783	445880	3,965
2		6,862	7722254	68,674
3		7,210	3076614	27,360

Table1, entry 4

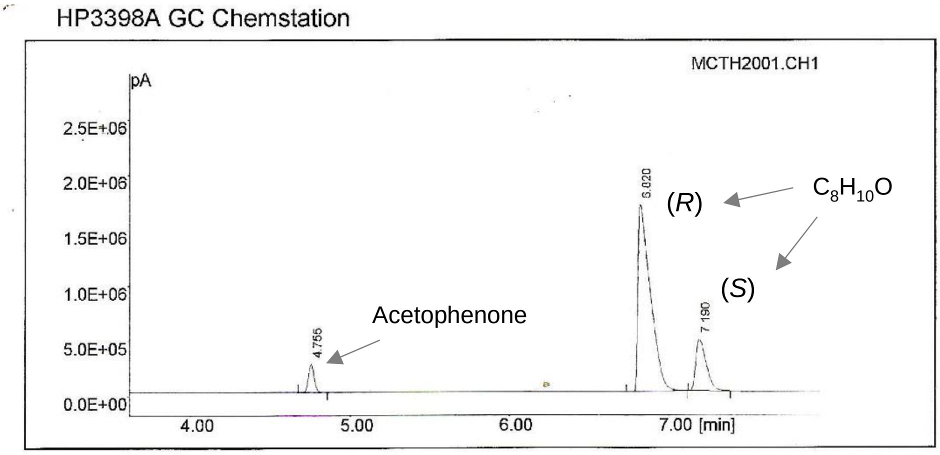

[RuCl$_2${PPh$_2$(OEt)}(^{i}Pr-pybox)] **(1c)**

Acetophenone/**1c**/KO^{t}Bu ratio = 500:1.24, ^{i}PrOH 50 mL

Time of reaction: 15 min

#	Nombre	Tr [min]	Área [pA*s]	%Área
1		4,755	608991	5,278
2		6,820	8874213	76,916
3		7,190	2054266	17,805

Table1, entry 5

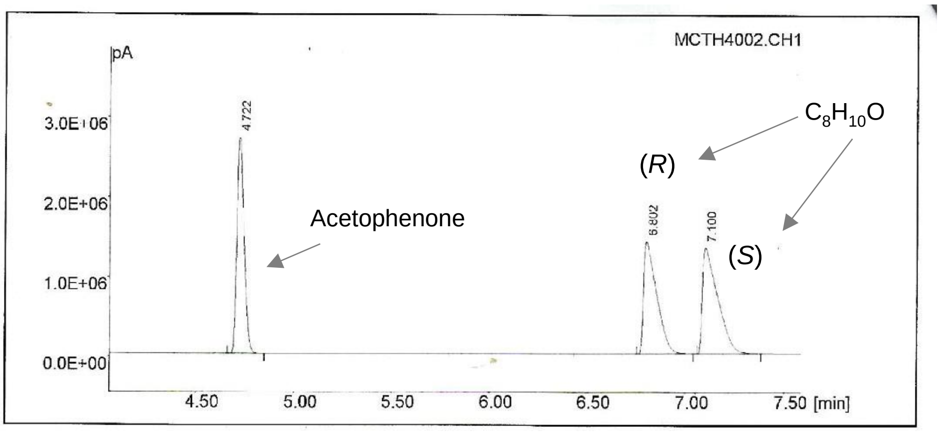

[$RuCl_2${PPh($OMe)_2$}(Ph-pybox)] **(2a)**

Acetophenone/**2a**/KO^{t}Bu ratio = 500:1.24, ^{i}PrOH 50 mL

Time of reaction: 15 min

#	Nombre	Tr [min]	Área [pA*s]	%Área
1		4,722	6532346	32,758
2		6,802	6356395	31,876
3		7,100	7052298	35,366

Table1, entry 6

HP3398A GC Chemstation

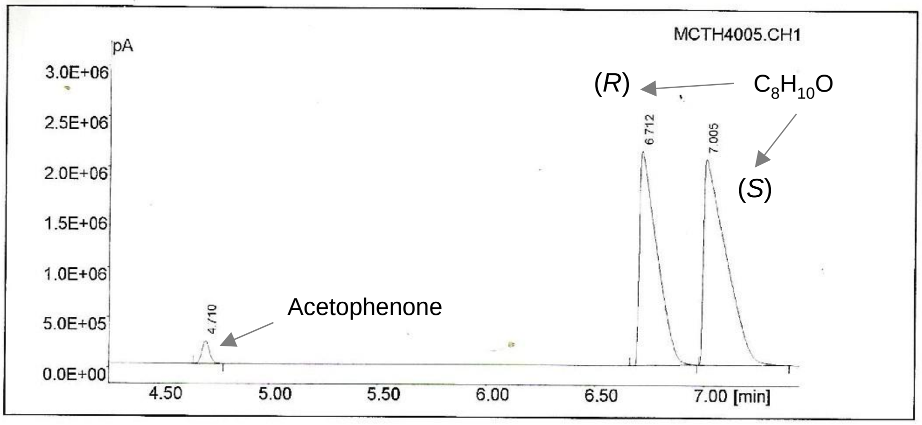

[RuCl$_2${PPh(OMe)$_2$}(Ph-pybox)] **(2a)**

Acetophenone/**2a**/KO^{t}Bu ratio = 500:1.24, ^{i}PrOH 50 mL

Time of reaction: 90 min

#	Nombre	Tr [min]	Área [pA*s]	%Área
1		4,710	520619	1,975
2		6,712	11558539	43,857
3		7,005	14276047	54,168

Table1, entry 7

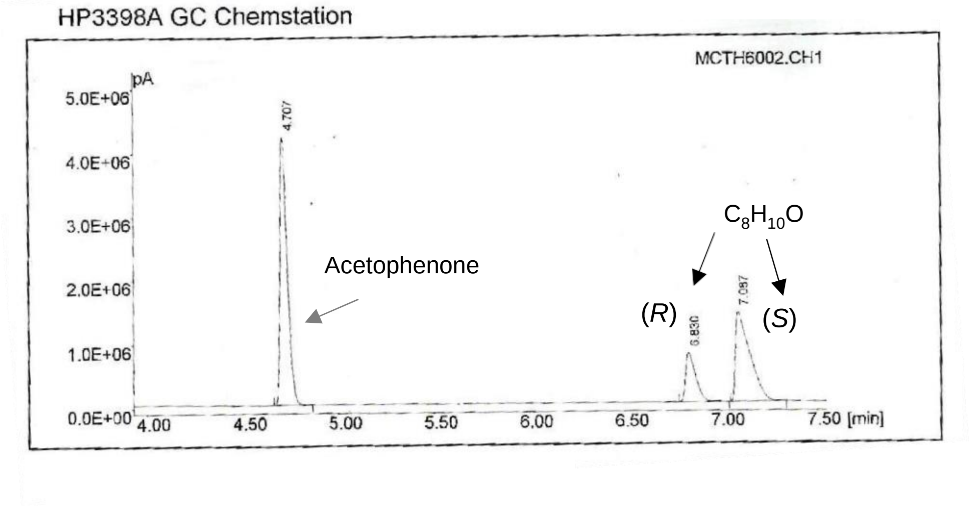

[$RuCl_2${PPh_2(OMe)}(Ph-pybox)] **(2b)**

Acetophenone/**2b**/KO^{t}Bu ratio = 500:1.24, ^{i}PrOH 50 mL

Time of reaction: 15 min

#	Nombre	Tr [min]	Área [pA*s]	%Área
1		4,707	10837275	52,466
2		6,830	2663075	12,893
3		7,087	7155435	34,641

Table1, entry 8

HP3398A GC Chemstation

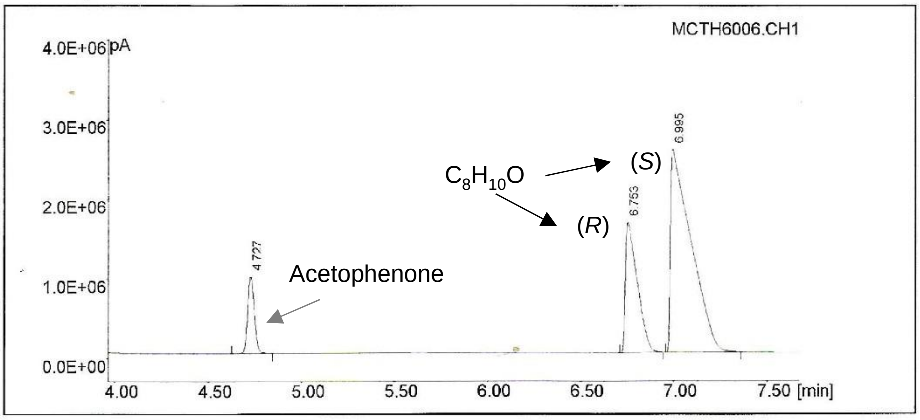

[$RuCl_2${$PPh(OMe)_2$}(Ph-pybox)] **(2b)**

Acetophenone/**2b**/KO^{t}Bu ratio = 500:1.24, ^{i}PrOH 50 mL

Time of reaction: 90 min

#	Nombre	Tr [min]	Área [pA*s]	%Área
1		4,727	2244862	7,905
2		6,753	7058183	24,854
3		6,995	19095530	67,241

Table1, entry 9

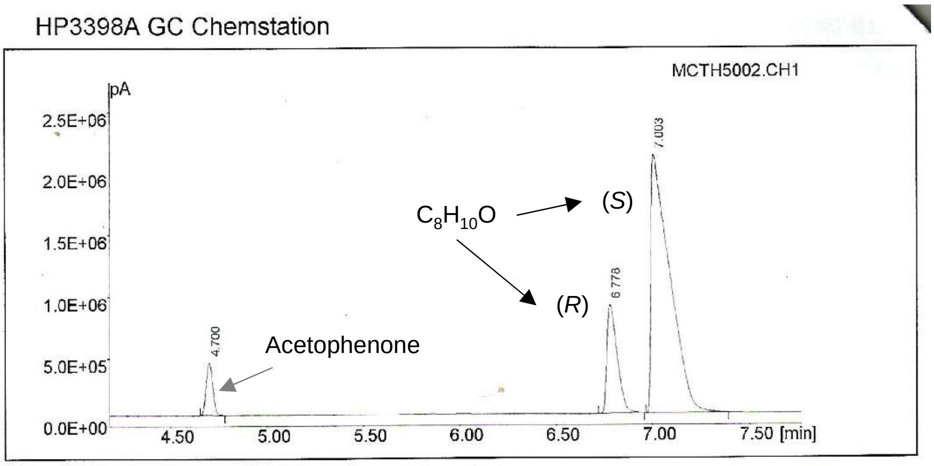

[RuCl$_2${PPh$_2$(OEt)}(Ph-pybox)] **(2c)**

Acetophenone/**2c**/KO^{t}Bu ratio = 500:1.24, ^{i}PrOH 50 mL

Time of reaction: 15 min

#	Nombre	Tr [min]	Área [pA*s]	%Área
1		4,700	955284	5,326
2		6,778	3131233	17,458
3		7,003	13849066	77,216

Table 2. Optimization of Reaction Conditions for Transfer Hydrogenation of Acetophenone Catalyzed by Ruthenium Complex [$RuCl_2${PPh_2(OEt)}(Ph-pybox)] (**2c**).

Enantiomeric excess: Determined by GC with a Supelco β-DEX 120 chiral capillary column.

Table 2, entry 1

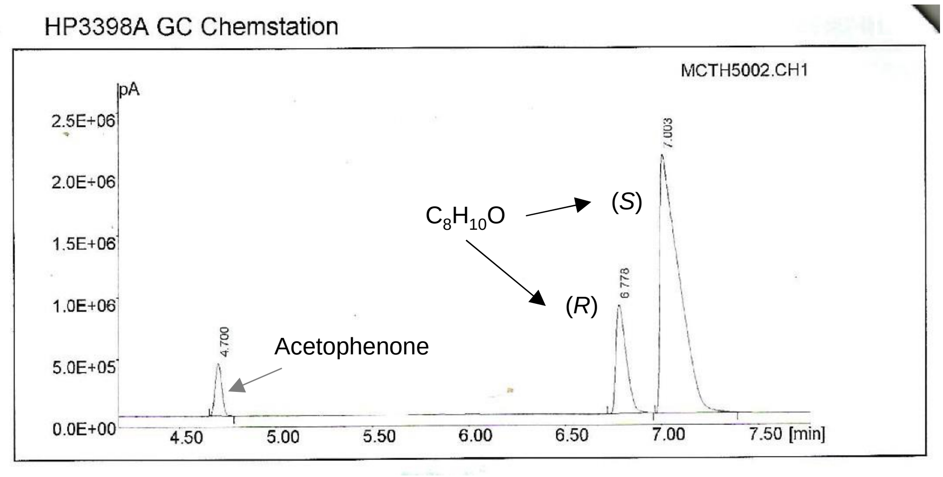

[$RuCl_2${PPh_2(OEt)}(Ph-pybox)] **(2c)**

Acetophenone/**2c**/KO^{t}Bu ratio = 500:1.24, ^{i}PrOH 50 mL
Time of reaction: 15 min

#	Nombre	Tr [min]	Área [pA*s]	%Área
1		4,700	955284	5,326
2		6,778	3131233	17,458
3		7,003	13849066	77,216

Table 2, entry 2

HP3398A GC Chemstation

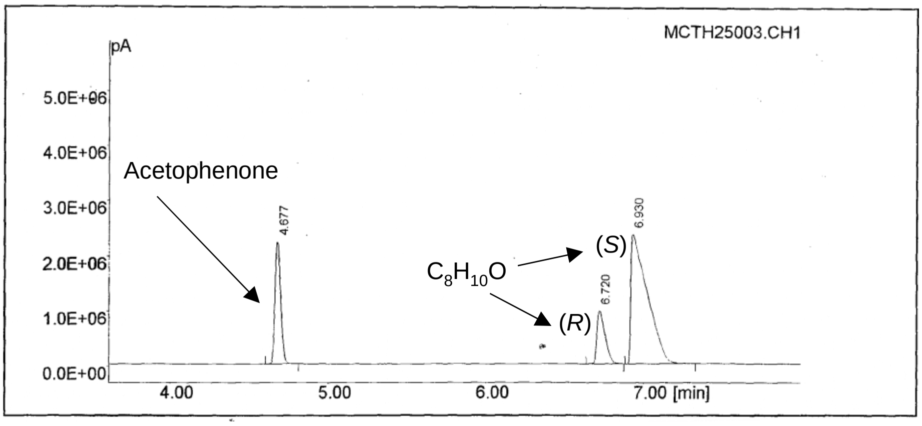

[RuCl$_2${PPh$_2$(OEt)}(Ph-pybox)] **(2c)**

Acetophenone/**2c**/KO^{t}Bu ratio = 500:0.5.24, ^{i}PrOH 50 mL

Time of reaction: 30 min

#	Nombre	Tr [min]	Área [pA*s]	%Área
1		4,677	5232233	20,819
2		6,720	3322629	13,221
3		6,930	16577363	65,961

Table 2, entry 3

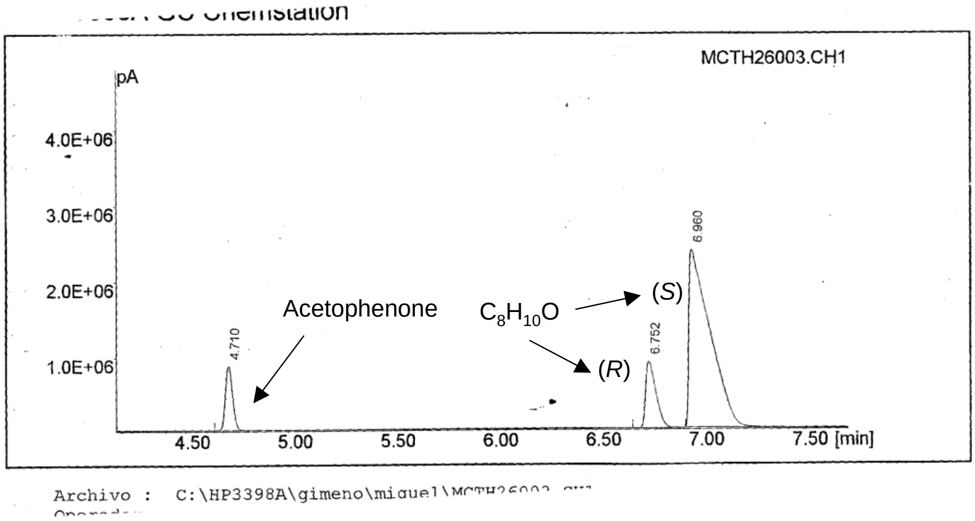

[RuCl$_2${PPh$_2$(OEt)}(Ph-pybox)] **(2c)**

Acetophenone/**2c**/KO^{t}Bu ratio = 500:1.5.24, ^{i}PrOH 50 mL
Time of reaction: 15 min

#	Nombre	Tr [min]	Área [pA*s]	%Área
1		4,710	1953066	9,167
2		6,752	2943484	13,816
3		6,960	16408554	77,017

Table 2, entry 4

HP3398A GC Chemstation

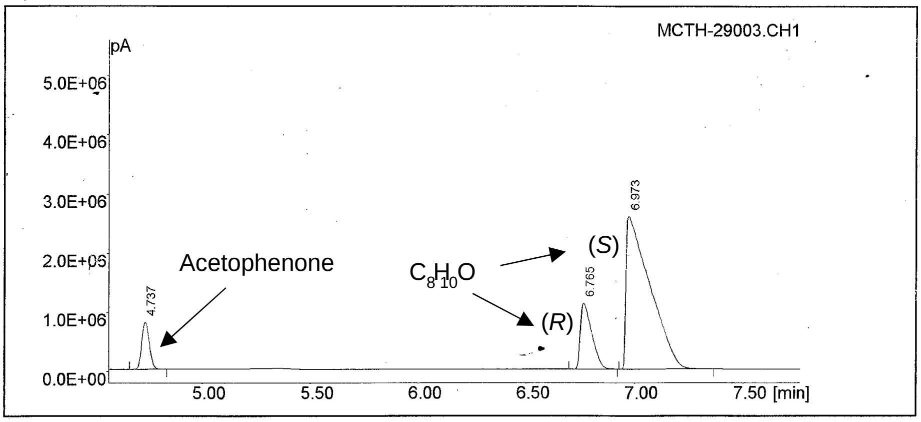

[$RuCl_2${PPh_2(OEt)}(Ph-pybox)] **(2c)**

Acetophenone/**2c**/KO^{t}Bu ratio = 500:2:24, ^{i}PrOH 50 mL

Time of reaction: 15 min

#	Nombre	Tr [min]	Área [pA*s]	%Área
1		4,737	1817967	7,207
2		6,765	3935930	15,603
3		6,973	19472220	77,191

Table 2, entry 5

HP3398A GC Chemstation

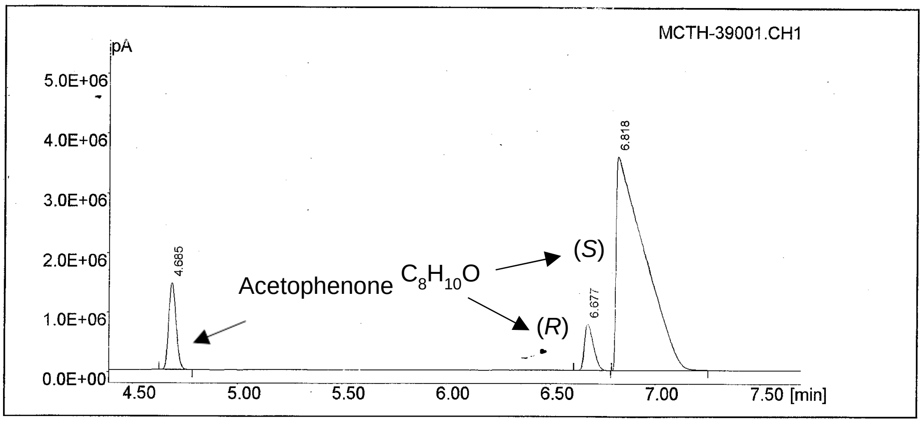

[$RuCl_2${PPh_2(OEt)}(Ph-pybox)] **(2c)**

Acetophenone/**2c**/KO^{t}Bu ratio = 500:1.5:24, ^{i}PrOH 75 mL
Time of reaction: 5 min

#	Nombre	Tr [min]	Área [pA*s]	%Área
1		4,685	3370756	8,648
2		6,677	2228378	5,717
3		6,818	33377300	85,635

Table 2, entry 6

HP3398A GC Chemstation

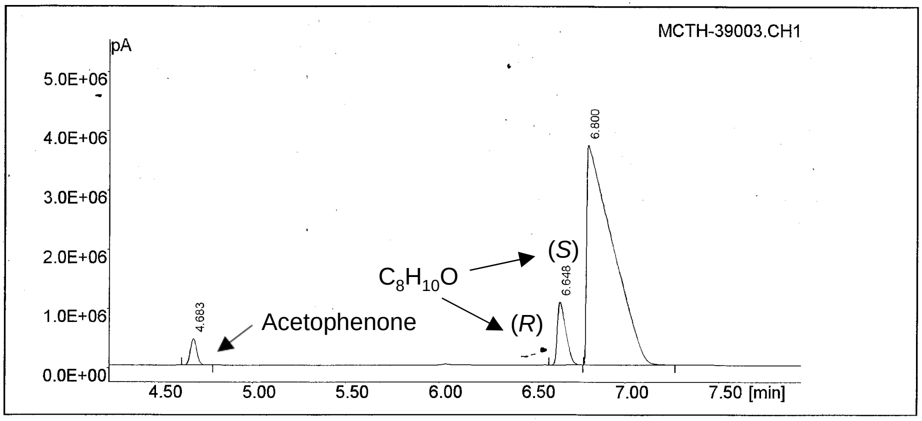

[$RuCl_2${PPh_2(OEt)}(Ph-pybox)] **(2c)**

Acetophenone/**2c**/KO^{t}Bu ratio = 500:1.5:24, ^{i}PrOH 75 mL
Time of reaction: 15 min

#	Nombre	Tr [min]	Área [pA*s]	%Área
1		4,683	992742	2,530
2		6,648	3214825	8,194
3		6,800	35026004	89,276

Table 2, entry 7

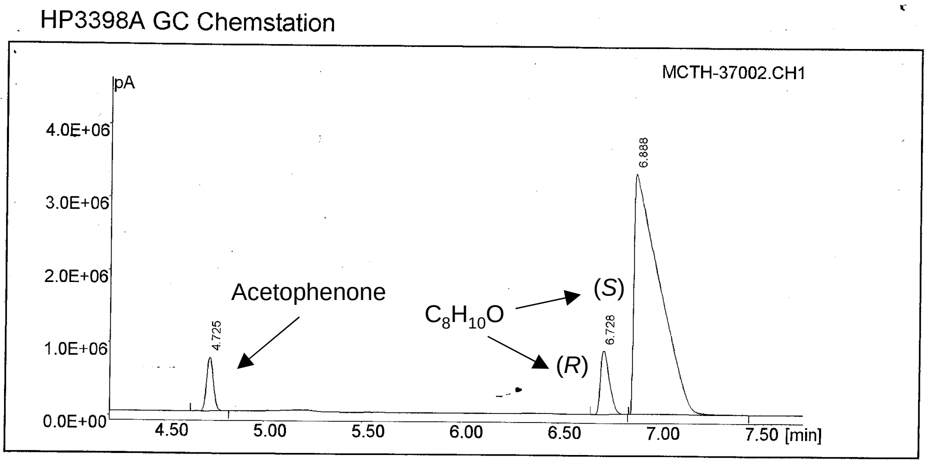

[RuCl$_2${PPh$_2$(OEt)}(Ph-pybox)] **(2c)**

Acetophenone/**2c**/NaOH ratio = 500:1.5.24, ^{i}PrOH 75 mL

Time of reaction: 10 min

#	Nombre	Tr [min]	Área [pA*s]	%Área
1		4,725	1680110	5,021
2		6,728	2656511	7,940
3		6,888	29122033	87,039

Table 2, entry 8

HP3398A GC Chemstation

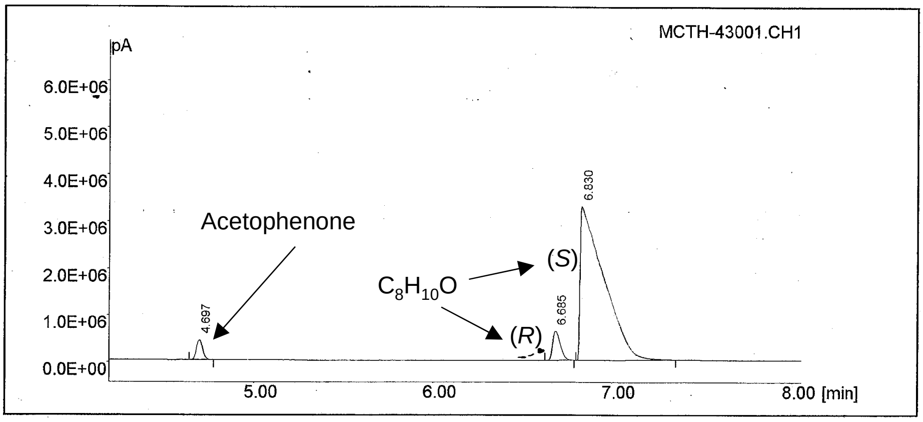

[RuCl$_2${PPh$_2$(OEt)}(Ph-pybox)] **(2c)**

Acetophenone/**2c**/NaO^{t}Bu ratio = 500:1.5:24, ^{i}PrOH 75 mL

Time of reaction: 5 min

#	Nombre	Tr [min]	Área [pA*s]	%Área
1		4,697	965350	2,999
2		6,685	1766683	5,489
3		6,830	29453028	91,511

Table 2, entry 9

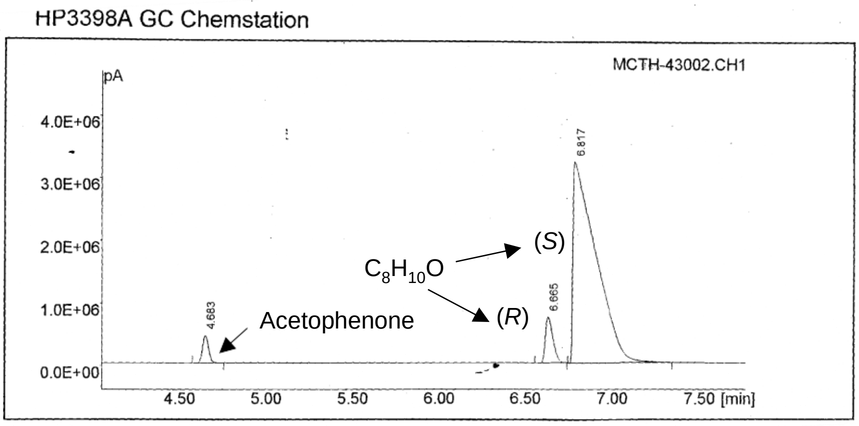

[RuCl$_2${PPh$_2$(OEt)}(Ph-pybox)] **(2c)**

Acetophenone/**2c**/NaO^{t}Bu ratio = 500:1.5:24, ^{i}PrOH 75 mL
Time of reaction: 10 min

#	Nombre	Tr [min]	Área [pA*s]	%Área
1		4,683	1004391	3,109
2		6,665	2152009	6,660
3		6,817	29154098	90,231

Table 2, entry 10

HP3398A GC Chemstation

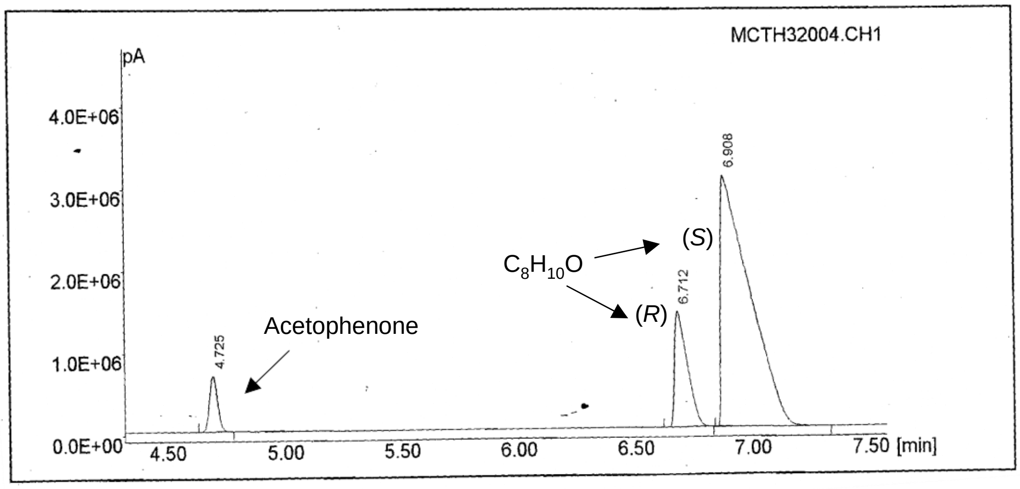

Archivo : C:\HP3398A\gimeno\miguel\MCTH32004.CH1
Operador : MIGUEL

[$RuCl_2${PPh_2(OEt)}(Ph-pybox)] **(2c)**

Acetophenone/**2c**/KOH ratio = 500:1.5.24, ^{i}PrOH 75 mL

Time of reaction: 30 min

#	Nombre	Tr [min]	Área [pA*s]	%Área
1		4,725	1538454	4,728
2		6,712	5175520	15,905
3		6,908	25826637	79,367

Table 2, entry 11

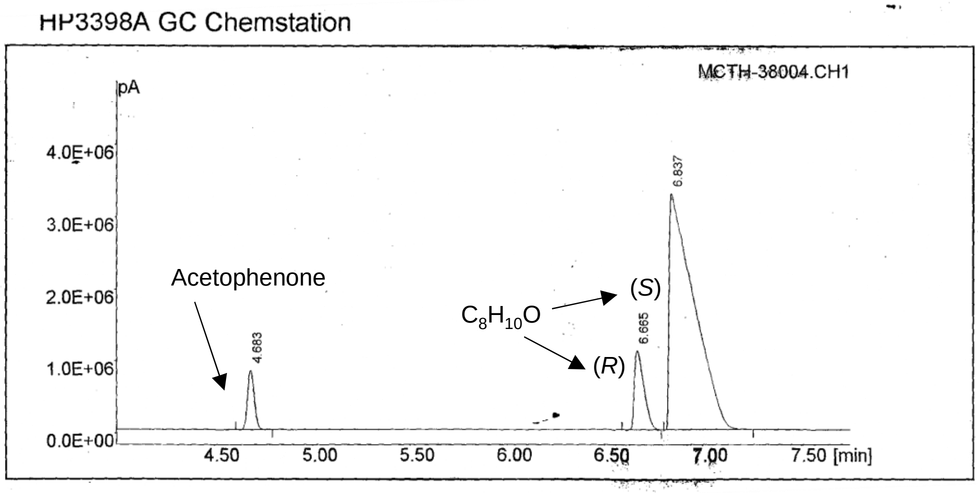

[$RuCl_2${PPh_2(OEt)}(Ph-pybox)] **(2c)**

Acetophenone/**2c**/Cs_2CO_3 ratio = 500:1.5:24, ^{i}PrOH 75 mL

Time of reaction: 30 min

#	Nombre	Tr [min]	Área [pA*s]	%Área
1		4,683	1858267	5,513
2		6,665	3580387	10,623
3		6,837	28265636	83,864

Table 2, entry 12

HP3398A GC Chemstation

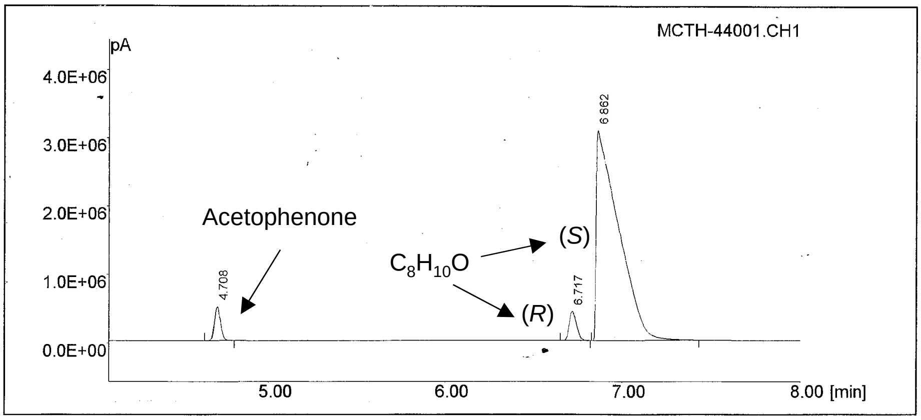

[RuCl$_2${PPh$_2$(OEt)}(Ph-pybox)] **(2c)**

Acetophenone/**2c**/NaO^{t}Bu ratio = 500:1.5:12, ^{i}PrOH 75 mL

Time of reaction: 5 min

#	Nombre	Tr [min]	Área [pA*s]	%Área
1		4,708	1128670	3,937
2		6,717	1163226	4,057
3		6,862	26379088	92,006

Table 2, entry 13

HP3398A GC Chemstation

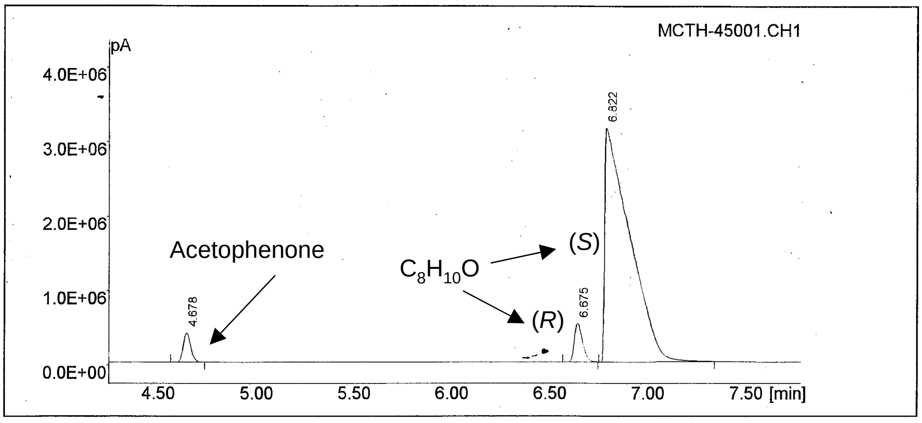

[$RuCl_2${PPh_2(OEt)}(Ph-pybox)] **(2c)**

Acetophenone/**2c**/NaO^{t}Bu ratio = 500:1.5:6, ^{i}PrOH 75 mL

Time of reaction: 5 min

#	Nombre	Tr [min]	Área [pA*s]	%Área
1		4,678	962076	3,232
2		6,675	1463890	4,917
3		6,822	27343562	91,851

Table 3. Transfer Hydrogenation of Ketones Catalyzed by Complex $[RuCl_2\{PPh_2(OEt)\}(Ph\text{-}pybox)]$ (**2c**) under Optimized Conditions.

Enantiomeric excess: Determined by GC with a Supelco β-DEX 120 chiral capillary column.

Table 3, entry 1

HP3398A GC Chemstation

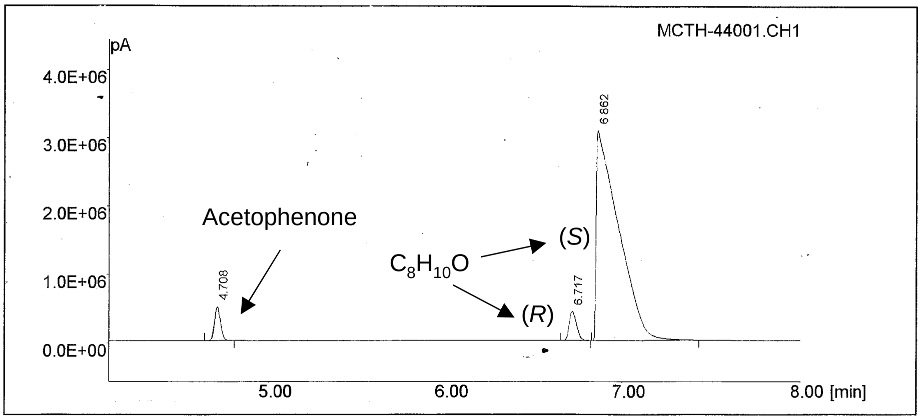

$[RuCl_2\{PPh_2(OEt)\}(Ph\text{-}pybox)]$ **(2c)**

Acetophenone/**2c**/NaO^{t}Bu ratio = 500:1.5:12, ^{i}PrOH 75 mL
Time of reaction: 5 min

#	Nombre	Tr [min]	Área [pA*s]	%Área
1		4,708	1128670	3,937
2		6,717	1163226	4,057
3		6,862	26379088	92,006

Table 3, entry 2

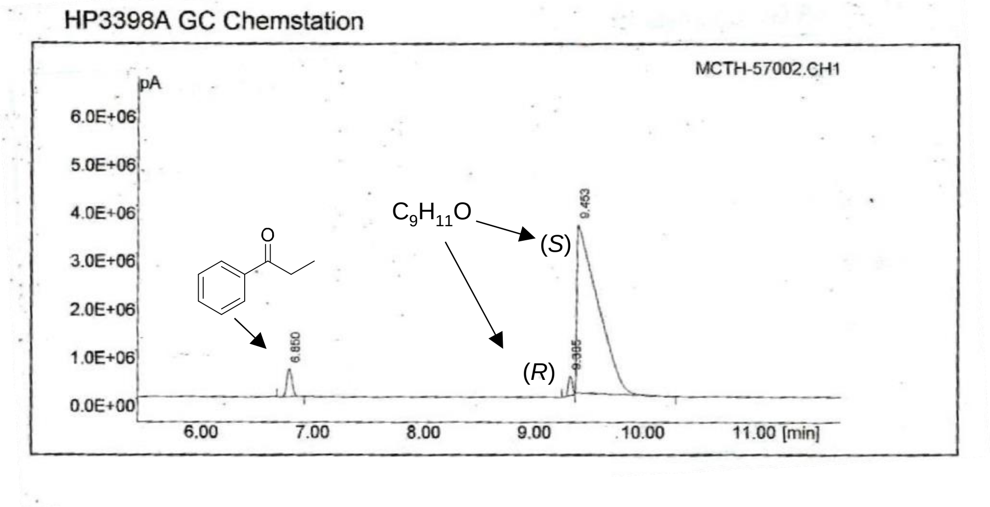

[RuCl$_2${PPh$_2$(OEt)}(Ph-pybox)] **(2c);** 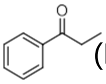 (Ketone)

Ketone/**2c**/NaO^{t}Bu ratio = 500:1.5:12, ^{i}PrOH 75 mL

Time of reaction: 10 min

#	Nombre	Tr [min]	Área [pA*s]	%Área
1		6,850	1755325	3,689
2		9,385	934146	1,963
3		9,453	44898518	94,348

Table 3, entry 3

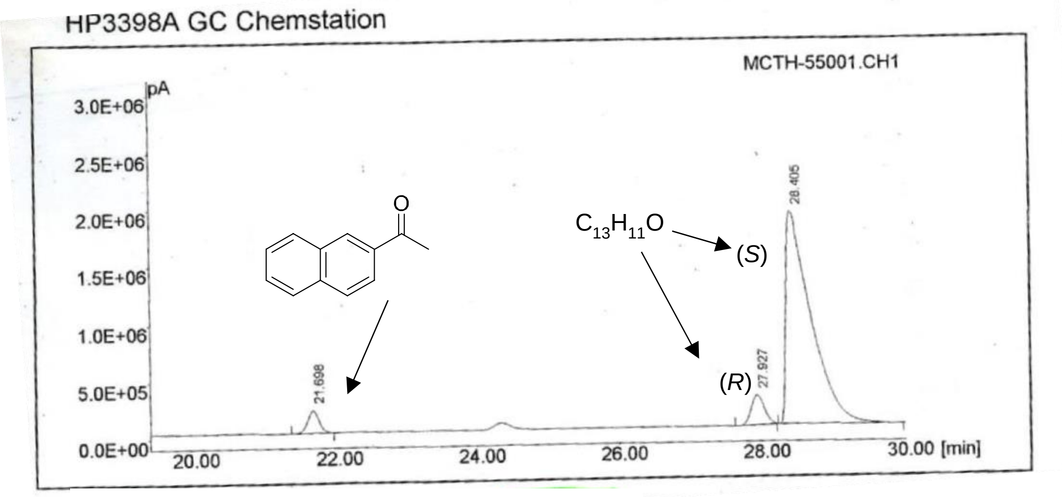

[RuCl$_2${PPh$_2$(OEt)}(Ph-pybox)] **(2c);** 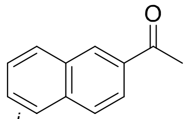 (Ketone)

Ketone/**2c**/NaO^{t}Bu ratio = 500:1.5:12, ^{i}PrOH 75 mL

Time of reaction: 5 min

#	Nombre	Tr [min]	Área [pA*s]	%Área
1		21,698	2081563	4,182
2		27,927	3221895	6,472
3		28,405	44476592	89,346

Table 3, entry 4

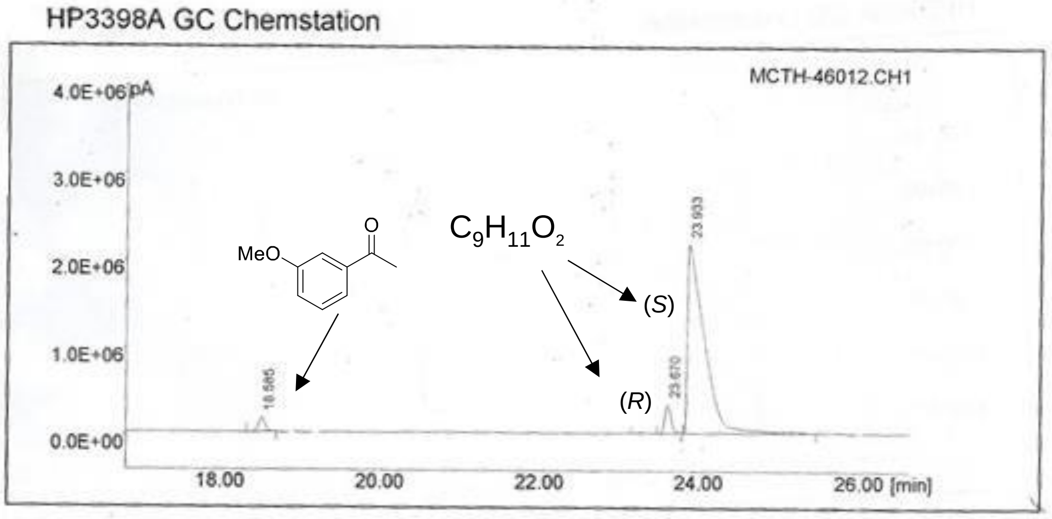

[RuCl$_2${PPh$_2$(OEt)}(Ph-pybox)] **(2c);** 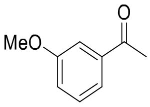

(Ketone)

Ketone/**2c**/NaO^{t}Bu ratio = 500:1.5:12, ^{i}PrOH 75 mL

Time of reaction: 5 min

#	Nombre	Tr [min]	Área [pA*s]	%Área
1		18,585	851791	2,544
2		23,670	1681749	5,022
3		23,933	30954371	92,434

Table 3, entry 5

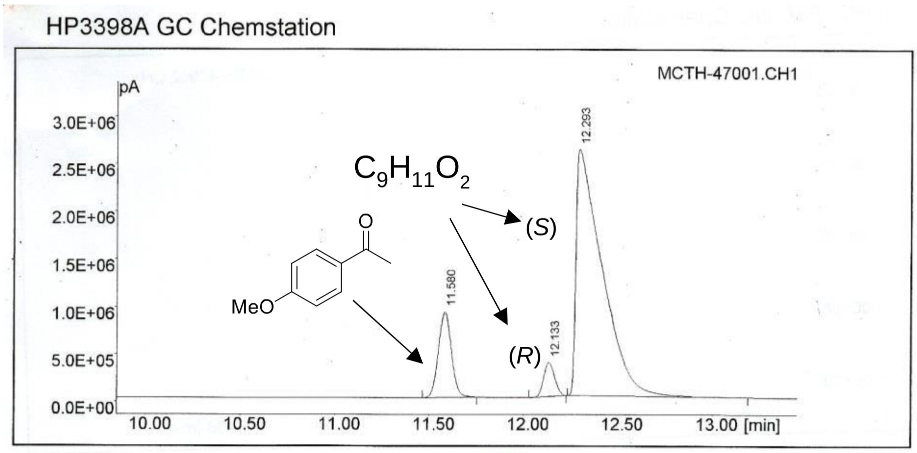

[RuCl$_2${PPh$_2$(OEt)}(Ph-pybox)] **(2c);** 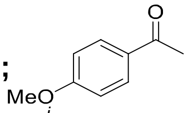 (Ketone)

Ketone/**2c**/NaO^{t}Bu ratio = 500:1.5:12, ^{i}PrOH 75 mL

Time of reaction: 5 min

#	Nombre	Tr [min]	Área [pA*s]	%Área
1		11,580	4235545	13,959
2		12,133	1430815	4,716
3		12,293	24675513	81,325

Table 3, entry 6

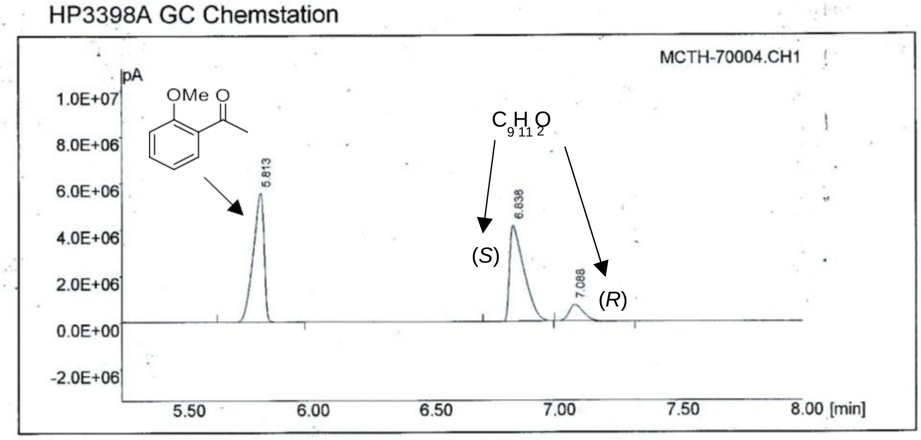

[RuCl$_2${PPh$_2$(OEt)}(Ph-pybox)] **(2c);** 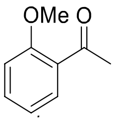 (Ketone)

Ketone/**2c**/NaO^{t}Bu ratio = 500:1.5:12, ^{i}PrOH 75 mL

Time of reaction: 45 min

#	Nombre	Tr [min]	Área [pA*s]	%Área
1		5,813	16910448	46,322
2		6,838	16797515	46,013
3		7,088	2798361	7,665

Table 3, entry 7

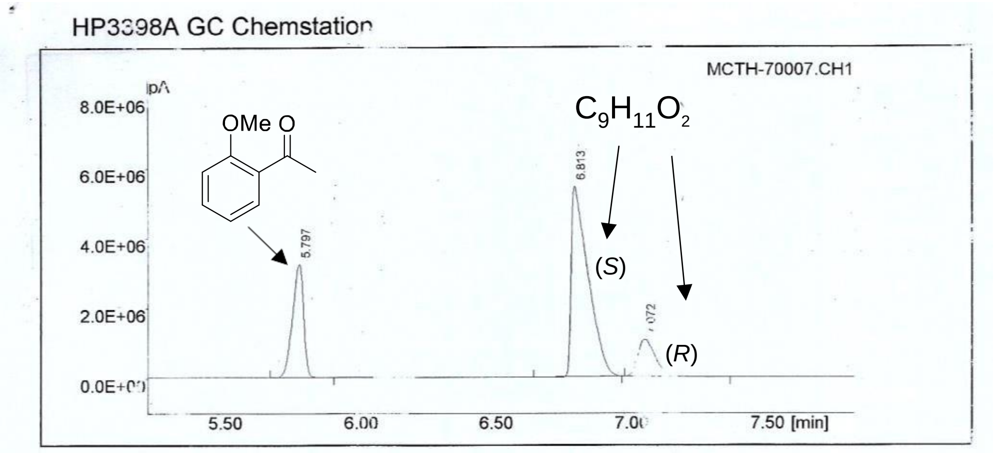

[RuCl$_2${PPh$_2$(OEt)}(Ph-pybox)] **(2c);** 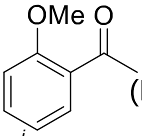 (Ketone)

Ketone/**2c**/NaO^{t}Bu ratio = 500:1.5:12, ^{i}PrOH 75 mL

Time of reaction: 90 min

#	Nombre	Tr [min]	Área [pA*s]	%Área
1		5,797	9199322	24,689
2		6,813	23516127	63,112
3		7,072	4545345	12,199

Table 3, entry 8

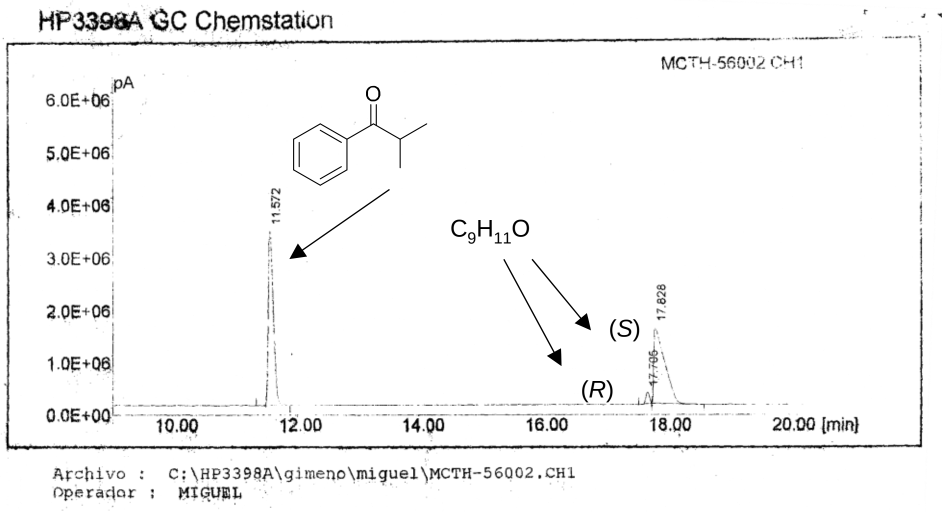

[RuCl$_2${PPh$_2$(OEt)}(Ph-pybox)] **(2c);** 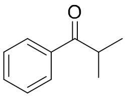 (Ketone)

Ketone/**2c**/NaO^{t}Bu ratio = 500:1.5:12, ^{i}PrOH 75 mL

Time of reaction: 10 min

#	Nombre	Tr [min]	Área [pA*s]	%Área
1		11,572	17733198	49,390
2		17,705	856177	2,385
3		17,828	17315334	48,226

Table 3, entry 9

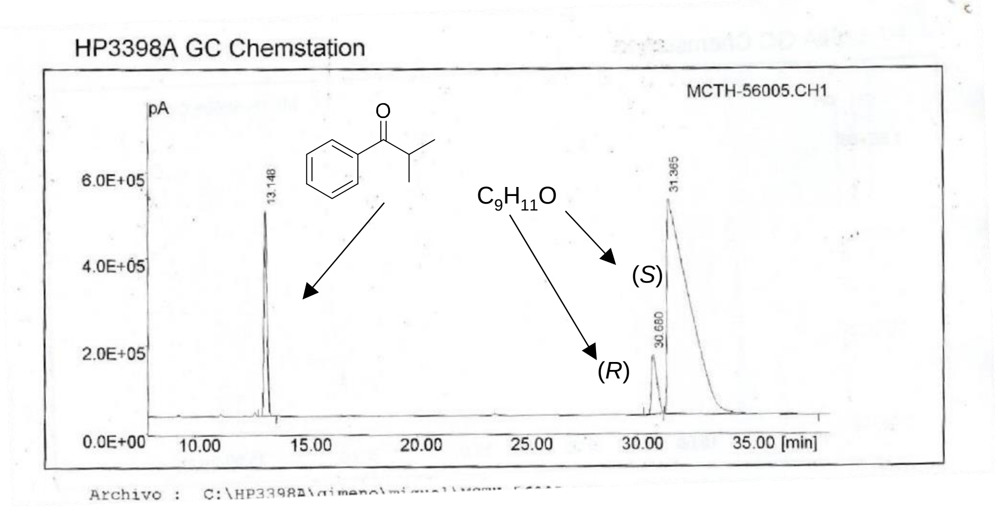

[RuCl$_2${PPh$_2$(OEt)}(Ph-pybox)] **(2c);** 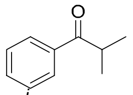 (Ketone)

Ketone/**2c**/NaO^{t}Bu ratio = 500:1.5:12, ^{i}PrOH 75 mL

Time of reaction: 60 min

#	Nombre	Tr [min]	Área [pA*s]	%Área
1		13,148	3799888	9,667
2		30,680	2294591	5,838
3		31,365	33212612	84,495

Table *S*1. Selected Bond Distances (Å) and Angles (deg) for Ruthenium(II) Complexes **1a**·$2CHCl_3$ and **2c**·$2.5CH_2Cl_2$

Complex **1a**·$2CHCl_3$			
Bond	Distance	Bond	Distance
Ru(1)–N(1)	2.078(6)	Ru(1)–Cl(1)	2.4126(15)
Ru(1)–N(2)	2.031(6)	Ru(1)–Cl(2)	2.4123(16)
Ru(1)–N(3)	2.089(6)	P(1)–O(3)	1.617(6)
Ru(1)–P(1)	2.2625(18)	P(1)–O(4)	1.611(5)
Angle	Value	Angle	Value
N(1)–Ru(1)–N(2)	77.1(3)	N(3)–Ru(1)–Cl(1)	90.97(17)
N(2)–Ru(1)–N(3)	77.3(3)	N(2)–Ru(1)–Cl(2)	85.25(18)
N(1)–Ru(1)–N(3)	154.4(2)	N(1)–Ru(1)–Cl(2)	91.80(18)
N(2)–Ru(1)–P(1)	178.3(2)	N(3)–Ru(1)–Cl(2)	86.71(17)
N(3)–Ru(1)–P(1)	101.16(17)	Cl(1)–Ru(1)–Cl(2)	171.60(6)
N(1)–Ru(1)–P(1)	104.45(18)	O(3)–P(1)–O(4)	103.5(3)
N(2)–Ru(1)–Cl(1)	86.37(18)	P(1)–Ru(1)–Cl(1)	92.93(6)
N(1)–Ru(1)–Cl(1)	86.80(18)	P(1)–Ru(1)–Cl(2)	95.44(6)
Complex **2c**·$2.5CH_2Cl_2$			
Bond	Distance	Bond	Distance
Ru(1)–N(1)	2.083(4)	Ru(1)–Cl(1)	2.3910(12)
Ru(1)–N(2)	2.020(4)	Ru(1)–Cl(2)	2.4032(12)
Ru(1)–N(3)	2.113(4)	P(1)–O(3)	1.621(4)
Ru(1)–P(1)	2.2831(13)		
Angle	Value	Angle	Value
N(1)–Ru(1)–N(2)	77.64(18)	N(3)–Ru(1)–Cl(1)	91.07(12)

N(2)–Ru(1)–N(3)	77.00(19)	N(2)–Ru(1)–Cl(2)	86.95(11)
N(1)–Ru(1)–N(3)	154.58(17)	N(1)–Ru(1)–Cl(2)	90.40(12)
N(2)–Ru(1)–P(1)	177.78(15)	N(3)–Ru(1)–Cl(2)	89.97(12)
N(3)–Ru(1)–P(1)	105.21(13)	Cl(1)–Ru(1)–Cl(2)	173.26(5)
N(1)–Ru(1)–P(1)	100.14(12)	P(1)–Ru(1)–Cl(1)	92.96(4)
N(2)–Ru(1)–Cl(1)	86.80(11)	P(1)–Ru(1)–Cl(2)	93.19(5)
N(1)–Ru(1)–Cl(1)	85.81(13)		

Table *S*2. Crystal Data and Structure Refinement for Complexes **1a**·$2CHCl_3$ and **2c**·$2.5CH_2Cl_2$

	**1a**·$2CHCl_3$	**2c**·$2.5CH_2Cl_2$
Empirical formula	$C_{27}H_{36}Cl_8N_3O_4PRu$·	$C_{39.5}H_{39}Cl_7N_3O_3PRu$
Formula weight	882.23	983.92
Temperature (K)	150(2)	150(2)
Wavelength (Å)	1.5418	1.5418
Crystal system	Monoclinic	Orthorhombic
Space group	$P2_1$	$P\ 2_12_12$
a (Å)	11.7788(2)	12.1533(1)
b (Å)	12.1699(1)	24.2508(2)
c (Å)	13.0210(2)	15.7095(2)
α (deg)	90	90
β (deg)	101.52(1)	90
γ (deg)	90	90
Z	4	4
Volume ($Å^3$)	1828.89(4)	4630.02(8)

$\rho_{calculated}$ (Mg m^{-3})	1.602	1.412
μ (mm^{-1})	9.567	7.090
F(000)	892	1996
Crystal size (mm^3)	0.04 x 0.06 x 0.12	0.01 x 0.06 x 0.47
θ range (deg)	3.46 to 69.65	2.813 to 69.627
Index ranges	$-14 \leq h \leq 11$	$-10 \leq h \leq 14$
	$-14 \leq k \leq 14$	$-29 \leq k \leq 28$
	$-15 \leq l \leq 13$	$-18 \leq l \leq 18$
No. of reflns. collected	15480	20835
No. of independent reflns.	6742 [R(int) = 0.0521]	8553 [R(int) = 0.0496]
Completeness to θ_{max} (%)	99.8	99.9
Refinement method	Full-matrix least-squares on F^2	Full-matrix least-squares on F^2
No.of parameters/restraints	403/0	493/0
Goodness-of-fit on F^2	1.048	1.065
Weight function (a, b)	0.0789, 0.7646	0.406, 0.8199
R [$I > 2\sigma(I)$]^a,	R_1 =0.0433, wR_2 = 0.1173	R_1 = 0.0353, wR_2 = 0.0857
R (all data)	R_1 =0.0454, wR_2 = 0.1205	R_1 = 0.0392, wR_2 = 0.0881
Absolute structure parameter	-0.019(7)	-0.015(6)
Largest diff. peak and hole (e Å^{-3})	1.666 and -0.938	0.507 and -0.514

^{a} $R_1 = \Sigma(|F_o| - |F_c|)/\Sigma|F_o|$; $wR_2 = \{\Sigma[w(F_o^2 - F_c^2)^2]/\Sigma[w(F_o^2)^2]\}^{½}$.

-
